# Cervical cancer screening and adherence to follow-up among Hispanic women study protocol: a randomized controlled trial to increase the uptake of cervical cancer screening in Hispanic women

**DOI:** 10.1186/1471-2407-12-170

**Published:** 2012-05-06

**Authors:** Catherine Duggan, Gloria Coronado, Javiera Martinez, Theresa L Byrd, Elizabeth Carosso, Cathy Lopez, Maria Benavides, Beti Thompson

**Affiliations:** 1 Public Health Sciences, Fred Hutchinson Cancer Research Centre, Seattle, WA, 98109, USA; 2 Kaiser Permanente Center for Health Research, Portland, OR, USA; 3 Department of Health Services, University of Washington, Seattle, WA, USA; 4 Department of Family Medicine Pontificia, Universidad Catolica de Chile, Santiago, Chile; 5 Texas Tech Paul L Foster School of Medicine, El Paso, TX, USA; 6 Breast, Cervical and Colon Health Program of Yakima Valley, Yakima, WA, USA; 7 Yakima Valley Farmworkers Clinic, Yakima, WA, USA

**Keywords:** Hispanic women, Pap test, Cervical cancer screening, Cancer disparities

## Abstract

**Background:**

In the US, Hispanic women have a higher incidence of, and mortality from, cervical cancer than non-Hispanic white women. The reason for this disparity may be attributable to both low rates of screening and poor adherence to recommended diagnostic follow-up after an abnormal Pap test. The 'Cervical Cancer Screening and Adherence to Follow-up Among Hispanic Women' study is a collaboration between a research institution and community partners made up of members from community based organizations, the Yakima Valley Farm Workers Clinic and the Breast, Cervical, and Colon Health Program of the Yakima District . The study will assess the efficacy of two culturally-appropriate, tailored educational programs designed to increase cervical cancer screening among Hispanic women, based in the Yakima Valley, Washington, US.

**Methods/design:**

A parallel randomized-controlled trial of 600 Hispanic women aged 21–64, who are non-compliant with Papanicolau (Pap) test screening guidelines. Participants will be randomized using block randomization to (1) a control arm (usual care); (2) a low-intensity information program, consisting of a Spanish-language video that educates women on the importance of cervical cancer screening; or (3) a high-intensity program consisting of the video plus a *‘promotora’* or lay-community health educator-led, home based intervention to encourage cervical cancer screening. Participants who attend cervical cancer screening, and receive a diagnosis of an abnormal Pap test will be assigned to a patient navigator who will provide support and information to promote adherence to follow-up tests, and any necessary surgery or treatment. Primary endpoint: Participants will be tracked via medical record review at community-based clinics, to identify women who have had a Pap test within 7 months of baseline assessment. Medical record reviewers will be blinded to randomization arm. Secondary endpoint: An evaluation of the patient navigator program as a method to improve adherence and reduce time to follow-up among participants who receive an abnormal Pap test result. An additional secondary endpoint is the cost-effectiveness of the two different intensity intervention programs.

**Discussion:**

This culturally sensitive intervention aims to increase compliance and adherence to cervical screening in a Hispanic population. If effective, such interventions may reduce incidence of cervical cancer.

**Trial registration:**

NCT01525433

## Background

Cervical cancer screening detects pre-cancerous changes in the cervix, such as cervical intraepithelial neoplasia (CIN) or cervical dysplasia. Introduction of the Papanicolau (Pap) test screening programs to women in all populations reduces cervical cancer rates by 60–90 percent within 3 years of implementation, with the reduction of mortality and morbidity consistent across populations [1,2]. In the US, the US Preventative Task Force guidelines for Pap testing are every 3 years for routine screening by women over the age of 21, or from three years after the age of initiation of sexual activity, whichever is earlier [3].

Hispanic women in the US have the highest cervical cancer incidence with an age-adjusted incidence of 12.5 cases/100,000 women for 2004–2008, compared to an incidence of 7.0 in the non-Hispanic white population [4]. Mortality from cervical cancer is also higher among Hispanics compared to non-Hispanic whites (estimates for age-adjusted U.S. mortality rates for 2008: 2.9 per 100,000 vs. 2.1 per 100,000) [4]. The disproportionate burden of cervical cancer among Hispanic women is thought to be attributable in part to both low rates of screening and poor adherence to recommended diagnostic follow-up after an abnormal Pap test. Data from the National Health Interview Survey show that 74.6% of Hispanic women had a Pap test in the past three years compared to 81.4% of non-Hispanic whites [5]. Similarly, Hispanic non-adherence with recommended follow-up has been reported in several regional studies to range from 20 to 90% [6,7].

Sociodemographic factors associated with non-adherence to cervical cancer screening in this population include low income, lack of health insurance, limited access to healthcare services, lack of clinician recommendation, length of residency in the United States, limited English language proficiency, acculturation, and lack of awareness of risks associated with non-participation in cervical cancer screening programs [8-11].

The aims of the Cervical Cancer Screening and Adherence to Follow-up Among Hispanic Women study are threefold: (1) to determine the effect of two different intensity, culturally appropriate information based interventions on Pap test utilization, vs. control; (2) to determine whether a patient navigator program can improve adherence to follow-up after a diagnosis of an abnormal Pap test, in a sample of Hispanic women in Yakima and Franklin counties of Washington (WA) State, US; and (3) to assess the cost-effectiveness of each of the intervention programs.

The study will be implemented with the aid of the two community based partners, the Yakima Valley Farm Workers’ Clinic (YVFWC) which operates a network of 13 federally qualified health center clinics in Eastern Washington specializing in the delivery of primary care services to low-income Latinos; and the Breast, Cervical, and Colon Health Program of the Yakima District (BCCHP), a federally funded aid program, administered through the Washington State Department of Health, which provides assistance and resources for women to undergo Pap tests, and for women who receive a diagnosis of an abnormal Pap test.

BCCHP and YVFWC will work with the study investigators to identify women who are non-compliant with Pap testing to monitor uptake of Pap test screening among randomized women via medical record review, and to develop and pilot test a patient navigator program to improve adherence and reduce time to follow-up after an abnormal Pap test.

To aid with issues regarding cultural relevance, a community advisory board was appointed and regular meetings are scheduled between the investigators and community members.

## Methods/design

**Figure 1:**
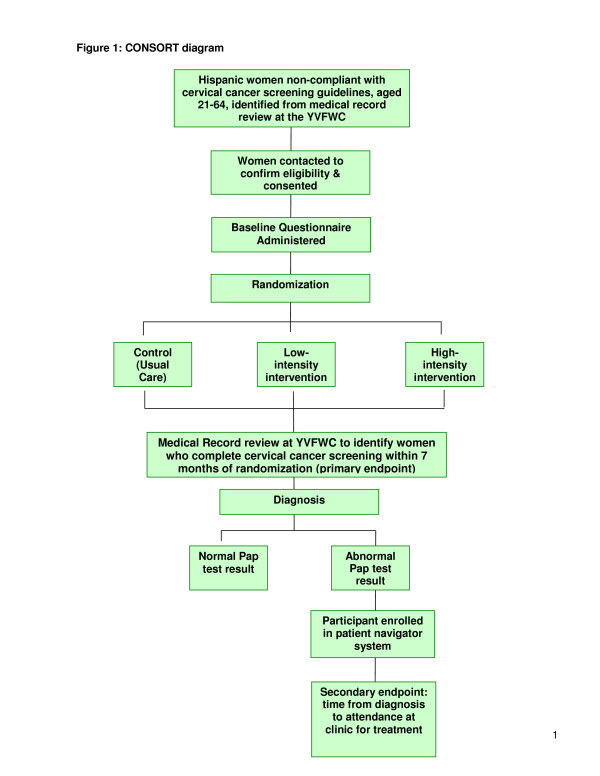
CONSORT diagram.

The ‘Cervical Cancer Screening and Adherence to Follow-up Among Hispanic Women’ study is a parallel randomized controlled trial with participants randomized into a control or usual care arm; a high intensity intervention *‘Promotora’* plus video arm; or a low-intensity ‘video’ only arm (Figure 1). The interventions are behavioural and information based, and are designed to educate participants about the importance of cervical cancer screening, and to encourage women to obtain screening. The study will be conducted in Yakima Valley, WA, US, and has been approved by the Institutional Review Board (IRB) of the Fred Hutchinson Cancer Research Center.

### Setting

In Washington State, much of the Hispanic population is concentrated in Yakima County, and Hispanics are the fastest growing population in the region, having increased by 37% between the 2000 and 2010 census, and the percentage of Hispanics is 45% of the population [12].

### Participants

The study aims to recruit 600 Hispanic women, who have been seen by a community health partner clinic (YVFWC) within the past 5 years, and who are out of compliance with current recommendations for Pap test screening, i.e., have not had a Pap test within the previous 3 years. Eligible women will be between 21 – 64 years of age as is consistent with guidelines, without a prior hysterectomy. Participants may be English or Spanish speakers and are resident in the Yakima Valley Washington, USA.

### Recruitment of participants

We will use recruitment strategies that were successfully used in our other studies involving community based clinics [13]. Details of Hispanic females who are eligible, who have been seen in the YVFWC clinics in the past five years, and who are non-compliant with cervical cancer screening recommendations will be extracted from computerized patient records. Eligible women will be invited to participate in the study by the YVFWC. Interested women will be told that they will be randomized to one of three arms in the project. The women will be assured that refusal to participate will not have any implications on the ability to receive care at the clinic. Women will be asked to sign an informed consent to participate in the project, and to sign a HIPAA authorization for release of medical record information for the purposes of verifying their use of cervical cancer screening. Once consent is obtained, a study staff member will telephone or go to the home of the participant to complete a baseline survey. The baseline questionnaire includes items on cervical cancer screening knowledge and attitudes as well as intention to be screened, acculturation, and sociodemographic variables.

### Randomization

Participants will be allocated to the intervention or control arms via a computerized program. A randomized block design assuring that randomization is balanced after every 15 participants will be used to ensure equal distribution across study arms. The randomization sequence was generated by a statistician who is not involved with the study. Medical record reviewers will be blinded to the randomization status of the participants.

### Interventions

Following baseline assessment, the participant will be randomized to control arm (usual care), mailed video only, or video plus *promotora* outreach, as described below.

#### Control arm

Participants who are allocated to the control arm (‘Usual Care’) will not receive any motivational messages or intervention materials from study staff. Usual care entails any information on the importance of regular Pap tests for cervical cancer risk reduction provided routinely by clinic staff to all women at community clinics.

#### Low intensity intervention (video)

Participants will be mailed a culturally-appropriate Spanish-language video with information and encouragement to undergo cervical cancer screening, information about resources to pay for screening, and other practical assistance such as resources for transportation. This intervention consists of a 13 minute video presentation of three different generational women talking in Spanish about their experiences and concerns for cervical cancer screening in the form of a “novela” or soap opera. The video has English subtitles for monolingual English-speaking participants. The video was developed as part of the ‘Ayudando a Las Mujeres con Informacion, Guia y Amor para su Salud’ (AMIGAS) intervention, which consisted of a lay community health educator or *promotora de salud*-(*promotora)* led in-home education session, and a video presentation. The culturally appropriate, theoretically-based curriculum was developed using a community-based participatory research approach, with the aim of increasing the uptake of cervical screening for Hispanic women living along the U.S./Mexican border [14].

#### High intensity intervention (video plus *promotora* outreach)

In this arm, women will receive a high-intensity intervention that includes *promotora*-led in-home education session. The educational session will include the culturally-appropriate video (as used in the low intensity intervention) [14], a “promise sheet” (i.e., a sheet that a woman signs to indicate what she will do next, such as thinking about having a Pap test, making an appointment), and a resource sheet which lists resources such as contact information for community resources for transportation, childcare, and other potential barriers for successful attendance at clinic. The *promotora* will have the opportunity to have a conversation about cervical cancer screening and directly schedule a Pap test for women through the clinic. For women who are not yet ready to schedule a Pap test, the *promotora* will make a telephone call to the woman two weeks later to review the action steps, and assess readiness to schedule a Pap test.

A *promotora* will be recruited by YVFWC from the community and will be provided with an initial training session lasting three days, which addresses procedures for approaching households and delivering the intervention, cervical cancer screening facts and tracking and documentation procedures. Other studies indicate *promotora* outreach to be a successful approach for Hispanic women [15-18].

Both the video and *promotora* led intervention are part of an educational curriculum created to increase cervical cancer screening in Hispanic women; the curriculum is based in Social Cognitive Theory (SCT). Definitions of social cognition vary, but central to the theory is that people’s social behavior can be understood by examining their beliefs about their behavior in a social context; and that people learn not only from their own experiences, but by observing the actions of others. According to SCT, three main factors, self-efficacy, goals and outcome expectancies, affect the likelihood of changing a health behavior [19,20].

### Primary outcome

The primary outcome is defined as completion of a Pap test within 7 months after baseline assessment. Participants will be tracked via medical record review at YVFWC. Differences in Pap testing rates in intervention and control arms will be assessed. Cost-effectiveness analysis will examine the associated costs per woman screened, by intervention arm.

### Secondary outcomes

As part of the reason for high cervical cancer mortality rates among Latinas is the lack of recommended follow-up, for abnormal Pap tests, a secondary outcome is compliance with such recommendations. The approach being used is a patient navigator approach where a *promotora* works closely with the participant to encourage her to attain the recommended follow-up.

The first patient navigator program was introduced by Dr. Harold Freeman, in a successful effort to increase access to early effective medical care for breast cancer patients at the Harlem Hospital in New York City [21]. Patient navigators aid patients in navigating barriers to receiving care, such as competing demands of work, family responsibilities, transportation and childcare. In addition, Hispanic women may experience specific barriers to care such as culturally specific health beliefs, immigration status and language barriers that would make obtaining and completing optimal care more difficult [22].

Following formative work interviewing Hispanic women in the Yakima Valley who had received abnormal Pap test results, we developed a culturally-appropriate patient navigator program. Any participant, who receives a diagnosis of an abnormal Pap test after their initial cervical cancer screening, will be offered the services of a patient navigator. The patient navigator will contact the participant and guide them through potential barriers to care, such as transportation, childcare, financial impediments and cultural or linguistic barriers. Patient navigator contact will cease when the participant has successfully completed follow-up diagnostic care or treatment. Patient navigators will be selected from the different community partner organizations to work collaboratively and ensure sustainability of the program. Training will provide basic knowledge on cervical cancer screening as well as communicational skills. The secondary outcome will measure the time between diagnosis of the first abnormal Pap test, and attendance at the clinic for follow up. Elapsed time in participants assigned to a patient navigator will be compared to a similar population of women seen in the clinic who are not part of the study.

### Sample size

Power is assessed for the primary endpoint, i.e. increased compliance with cervical cancer screening. We assume compliance probabilities will range from 0.15 to 0.30 for usual care, 0.30 to 0.45 for the low-intensity arm, and 0.45 to 0.60 for the high-intensity arm with at least a 0.15 difference between the low intensity and control arms and the low and high intensity arms. Assuming a 10% loss to follow-up these power calculations are based on a sample size of 180 in each of the three arms, assuming two Chi square tests will be conducted; one to compare the low intensity arm to the control arm and one to compare the high intensity arm to the control arm. The overall significance level for the two tests was targeted at 0.05 so that each test is powered with significance level 0.025 to account for the two primary evaluations.

### Statistical analysis

The primary end-point (Pap test compliance) will be coded as a binary variable. Evaluation of the success of the intervention is based only on Pap test compliance at follow-up. Chi square tests of 2 X 2 tables (compliance Yes/No by arm Control vs. High or Low intensity intervention) will be employed to assess whether intervention arms affects the probability of compliance at follow-up. We will randomize about 600 women (anticipated 200 per arm to each of 3 arms; control, low intensity intervention, high intensity intervention) into the study, which will allow for 10% attrition. Separate analysis will be conducted to compare the high intensity arm to the control arm and the low intensity arm to the control arm, and subsequent analysis will include comparing the low intensity arm to the high intensity arm as well as adjustment of the primary analysis by potential confounding characteristics such as race and age, to account for potential biases in the randomization.

Additional secondary analysis will include evaluating the effect of contact from a patient navigator to women who receive an abnormal Pap smear. All participants seen at the study clinics during the course of the study who receive an abnormal Pap smear (intervention population) will receive a contact from the patient navigator. These women will be compared to women who received an abnormal Pap smear at the same clinics between 180 and 90 days prior to the start of the study when no navigator calls were made (control population) as determined by medical records. The outcome measure will be determination of follow up for an abnormal Pap smear within 90 days of initial contact by the clinic. We will compare length of time from initial diagnosis to attendance at the clinic for recommended follow-up in the participants who have been contacted by the patient navigator, vs. the control population

## Discussion

The ‘Cervical Cancer Screening and Adherence to Follow-up among Hispanic Women’ study has the potential to identify mechanisms to reduce health disparities in cervical cancer incidence through increasing uptake of cancer screening and thereby increasing early detection. The findings of this study will help address the disparate burden of cervical cancer among Hispanic women by implementing an innovative program to improve rates of cervical cancer screening, and it is the joint expectation of the research team and the clinic system that the clinic will continue to use the most effective and cost-effective intervention as part of its usual service to women in need of cervical cancer screening. Evaluation of a patient navigator program to aid Hispanic women in overcoming obstacles to receive follow-up care in a timely manner after a diagnosis of an abnormal Pap test, may help to increase the proportion of women who receive timely follow-up care in health care clinics in rural Eastern Washington. If successful, the patient navigator system may represent a method of reducing cancer morbidity and mortality, by removing cultural and other barriers and thus allowing women to successfully access appropriate healthcare after a diagnosis of a cervical abnormality.

Finally, the evaluation of cost-effectiveness of the intervention arms will aid in the design of appropriate inventions where financial constraints in underfunded health care systems may be of concern.

## Abbreviations

YVFWC: Yakima Valley Farm Workers Clinic; BCCHP: Breast, Cervical and Colon Health Program of the Yakima District; HIPAA: Health Insurance Portability and Accountability Act of 1996; SCT: Social Cognitive Theory.

## Competing interests

The authors declare that they have no competing interests.

## Authors’ contributions

BT and GC designed the clinical trial. BT obtained funding and critically revised the manuscript. CD developed study questionnaires and protocols, and drafted the manuscript. JM carried out formative research into the clinical trial design provided expertise on cervical cancer screening and critically revised the manuscript. EC implemented the protocol and critically revised the manuscript. TB led the studies that originally developed and tested AMIGAS. JLB developed the intervention. CL and MB collaborated in the design and implementation of the project. All authors read and approved the final version of the manuscript.

## Pre-publication history

The pre-publication history for this paper can be accessed here:

http://www.biomedcentral.com/1471-2407/12/170/prepub
